# Heart rate variability, quality of life, and sleep quality in patients with epilepsy

**DOI:** 10.1590/1806-9282.20250002

**Published:** 2025-08-08

**Authors:** Selcen Duran, Yalcin Boduroglu, Asuman Celikbilek

**Affiliations:** 1 Kirsehir Ahi Evran University, Faculty of Medicine, Department of Neurology – Kirsehir, Turkey.; 2 Kirsehir Ahi Evran University, Faculty of Medicine, Department of Cardiology – Kirsehir, Turkey.

**Keywords:** Cognition, Epilepsy, Heart rate, Quality of life, Sleep quality

## Abstract

**OBJECTIVE:**

Recent data have shown that patients with epilepsy experience reduced quality of life and poor sleep quality, which are also closely related to heart rate variability. For the first time, we investigated the relationship between heart rate variability and quality of life and sleep in patients with epilepsy.

**METHODS:**

Twenty-seven patients with medically controlled epilepsy, 23 patients with drug-resistant epilepsy, and 36 healthy subjects were included in this cross-sectional prospective study. Heart rate variability analysis was conducted using a 24-h rhythm Holter device in all cases. The quality of life in epilepsy-31 questionnaire and Pittsburgh Sleep Quality Index questionnaire were used for patients with epilepsy.

**RESULTS:**

Compared to the control group, patients with epilepsy had lower heart rate variability parameters, including standard deviation of normal-to-normal, standard deviation of normal-to-normal index, standard deviation of the averages of the normal-to-normal, root mean square of successive differences, and percentage of NN Intervals >50 ms (pNN50) values (p<0.001). Heart rate variability parameters and sleep scores were similar between the drug-resistant epilepsy and medically controlled epilepsy subgroups (p>0.05). In addition, quality of life in epilepsy-31 subscores were significantly lower in drug-resistant epilepsy than in medically controlled epilepsy in the subdomains of seizure worry (p=0.001), overall quality of life (p=0.004), emotional well-being (p=0.005), and cognitive functioning (p=0.007). There was a significant positive correlation between standard deviation of the averages of the normal-to-normal and cognition (r=0.335; p=0.017); maximum QT and emotional well-being (r=0.286; p=0.046); and maximum QTc and emotional well-being (r=0.292; p=0.042) in patients with epilepsy.

**CONCLUSION:**

We found that low heart rate variability was more common in patients with epilepsy. Quality of life was also highly impaired in drug-resistant epilepsy, and low heart rate variability correlated with low cognition and emotional well-being in patients with epilepsy.

## INTRODUCTION

Epilepsy significantly impacts patients’ quality of life through seizure frequency, cognitive deficits, and psychiatric comorbidities, which are well-documented in the literature^1^. Among these, seizure frequency and/or severity and coexisting psychiatric problems are the most common^1^. Patients with epilepsy (PWE) also have poor sleep quality caused by sleep fragmentation due to frequent seizures or nocturnal attacks^2^. However, less attention has been given to the role of autonomic nervous system dysfunction, as reflected in heart rate variability (HRV), in shaping these outcomes. HRV analysis non-invasively detects autonomic dysfunction, with high HRV indicating vagal dominance and low HRV reflecting reduced vagal output^3^.

Recent studies have established that diminished HRV is associated with an increased risk of sudden unexpected death in epilepsy (SUDEP) and impaired cognitive function in other neurological disorders^3^. As well, HRV was shown to affect the quality of life^4^ and sleep pattern^5^. Despite this, the potential utility of HRV as a biomarker for quality of life and sleep quality in epilepsy remains uninvestigated.

This study pioneers the examination of HRV in relation to quality of life and sleep quality in PWE. By highlighting the interconnections between autonomic function, cognitive health, and emotional well-being, this work proposes a novel paradigm for managing epilepsy that integrates autonomic metrics into routine clinical practice. This innovative perspective not only bridges two critical disciplines—neurology and cardiology—but also opens avenues for improving long-term outcomes in epilepsy management.

## METHODS

Fifty PWE were recruited from the neurology outpatient clinic, and 36 age- and sex-matched healthy subjects, recruited from the hospital staff, ranging from 18 to 60 years old, were included in this cross-sectional prospective study. The criteria for these classifications were based on the International League Against Epilepsy (ILAE) guidelines. Patients with a history of cardiac disease, known sleep problems, mental retardation, other neurological or psychiatric comorbidities, systemic diseases such as hypertension, diabetes mellitus, cerebrovascular diseases, anemia, or those who were on antiarrhythmic medication were excluded from the study.

Demographic data and information about epileptic seizures, including the duration of epilepsy, seizure type and frequency, routine electroencephalography (EEG) findings, and the antiseizure medication, were documented. The PWE were divided into two primary groups: individuals with drug-resistant epilepsy (DRE) and individuals with medically controlled epilepsy (MCE). The ILAE defines DRE as the inability to attain sustained seizure freedom despite undergoing adequate trials of two tolerated, appropriately selected, and administered medication regimens, which may be utilized either as monotherapies or in combination^6^. Patients >1 year seizure-free on medication were considered MCE. Epileptic seizures were classified as partial, generalized, and unknown etiology according to the ILAE committee on diagnosis, classification, and terminology. Regarding seizure frequency, PWE were divided into three groups: seizures more often than one per month, seizures more often than one per year, and seizure-free for >1 year. According to EEG findings, PWE were divided into three groups as normal, focal findings, and generalized findings.

This study was conducted at the Department of Neurology, Kirsehir Ahi Evran University Training and Research Hospital, between February 2023 and October 2023 in accordance with the Declaration of Helsinki. Ethical approval for this study was obtained from the Kirsehir Ahi Evran University Ethics Committee in Kirsehir Ahi Evran University on the date of 09/07/2024, with approval number of 2024–13/105.

### Heart rate variability parameters

HRV analysis was conducted using a 24-h rhythm Holter device on all patients. It was recommended that patients should not be restricted in their daily life during enrollment. Recordings were analyzed both automatically and manually using the Holter analysis software of DMS CardioScan II, with the analysis conducted by the same cardiologist. HRV indices include standard deviation of normal-to-normal (SDNN), the standard deviation of all the normal-to-normal (NN) intervals for each 5 min segment of a 24-h HRV recording (SDNN index), the standard deviation of the average NN intervals for each of the 5 min segments (SDANN), root mean square of successive differences (RMSSD), pNN50, and corrected QT interval (QTc).

### Quality of life

The quality of life in epilepsy-31 (QOLIE-31) questionnaire was used to measure the quality of life in PWE. This questionnaire consists of 31 questions, scored from 0 to 100 points; a high score indicates a high quality of life. The questionnaire comprises seven sections, which include seizure worry, overall quality of life, emotional well-being, energy and fatigue, cognitive function, medication effects, social functioning, and a single item covering overall health^7^.

### Sleep quality

The Pittsburgh Sleep Quality Index (PSQI) questionnaire was utilized to evaluate sleep quality in PWE. It consists of 21 questions, and a high score indicates poor sleep quality. The cutoff value is five points^8^.

### Statistical analysis

Data were analyzed using International Business Machines (IBM) SPSS version 23. Compliance with the normal distribution was assessed using the Kolmogorov-Smirnov. The Mann-Whitney U test was used to compare non–normally distributed data between paired groups, while the independent two-sample t-test was used to compare normally distributed data. One way analysis of variance (ANOVA) was used to compare normally distributed data among groups of three or more, and multiple comparisons were analyzed using the Duncan test. The Kruskal-Wallis test was used to compare non–normally distributed data among groups of three or more, and multiple comparisons were analyzed using Dunn's test. The chi-square and Fisher-Freeman-Halton tests were used to compare categorical data across the different groups. Pearson's correlation coefficient was used to correlate normally distributed quantitative data, while Spearman's rho correlation coefficient was used to correlate non-normally distributed quantitative data. Analysis results were presented as mean±standard deviation, median (interquartile ranges), or frequency (percentage). The significance level was set at p<0.05.

## RESULTS

Demographic data and HRV parameters of PWE and the control group are summarized in Table 1. The age and gender distributions of the participants in the PWE and control groups were similar. In comparison to the control group, PWE exhibited lower values of SDNN, SDNN index, SDANN, RMSSD, and pNN50, while demonstrating higher minimum heart rate values (p<0.001).

**Table 1:** Demographic data and heart rate variability parameters in epilepsy patients and controls.

		PWE (n=50)	Controls (n=21)	p-value
Female gender		29 (58)	10 (47.6)	0.422
Age		37 (26–49.5)	35 (28–40)	0.673
HRV parameters				
	SDNN (ms)	123 (102–161.5)	164 (153.5–187)	**<0.001**
	SDNN index	53 (43.5–64.5)	73 (68–78)	**<0.001**
	SDANN (ms)	110 (90.5–145)	150 (136–176)	**<0.001**
	RMSSD (ms)	29 (21–38)	62 (58.5–67.5)	**<0.001**
	pNN50 (%)	7 (2–15)	22 (18–26)	**<0.001**
	Minimum HR (b/m)	52 (48–55.5)	44 (40.5–46)	**<0.001**
	Maximum HR (b/m)	158 (136–243)	152 (132.5–170.5)	0.325
	Average HR (b/m)	80 (75–88.5)	77 (71.5–83.5)	0.152
	Maximum QT (ms)	481 (454–583)	470 (444.5–631.5)	0.672
	Maximum QTc (ms)	544 (500–630)	554 (511.5–678)	0.442

Values are expressed as n (%) or median (25th–75th percentile). PWE: patients with epilepsy, HRV: heart rate variability, SDNN: standard deviation of RR intervals, SDNN index: the standard deviations of all the NN intervals for each 5 min segment of a 24-h HRV recording, SDANN: the standard deviation of the average NN intervals for each of the 5 min segments, RMSSD: root mean square of the successive differences of inter-beat intervals, pNN50: percentage of adjacent NN intervals differing by more than 50 milliseconds, HR: heart rate; QTc: corrected QT. p<0.05 was considered statistically significant.

Clinical data of DRE and MCE subgroups in PWE are shown in Table 2. While the epilepsy type and EEG findings did not significantly differ between DRE and MCE (p>0.05), the disease duration was significantly longer in DRE (p=0.019). Drug use was also significantly different between these subgroups. Valproic acid, lacosamide, and lamotrigine use was higher in the DRE group (p=0.009, p=0.010, p=0.024, respectively).

**Table 2:** Comparison of clinical features between medically controlled and drug-resistant epilepsy patients.

		MCE (n=27)	DRE (n=23)	p-value
Disease duration (years)		15.6±10.2	23±11.3	**0.019**
Seizure type				
	Focal	14 (51.9)	18 (78.3)	0.136
	Generalized	6 (22.2)	3 (13)	
	Unknown etiology	7 (25.9)	2 (8.7)	
EEG findings				0.693
	Normal	15 (55.6)	10 (43.5)	
	Focal findings	10 (37)	11 (47.8)	
	Generalized findings	2 (7.4)	2 (8.7)	
Antiseizure medication				
	Levetiracetam	13 (48.1)	17 (73.9)	0.064
	Valproic acid	3 (11.1)	10 (43.5)	**0.009**
	Carbamazepine	5 (18.5)	9 (39.1)	0.106
	Lacosamide	1 (3.7)	7 (30.4)	**0.010**
	Lamotrigine	4 (14.8)	10 (43.5)	**0.024**
	Topiramate	1 (3.7)	0 (0)	0.351
Seizure frequency				**<0.001**
	Seizure free	27 (100)	0 (0)	
	Seizures more often than 1 per month	0 (0)	7 (30.4)	
	Seizures more often than 1 per year	0 (0)	16 (69.6)	

Values are expressed as n (%) or mean±SD. MCE: medically controlled epilepsy, DRE: drug-resistant epilepsy, EEG: electroencephalography. p<0.05 was considered statistically significant.

HRV parameters, PSQI, and QOLIE-31 scores of DRE and MCE subgroups are presented in Table 3. HRV parameters and sleep scores were similar between the subgroups (p>0.05). QOLIE-31 total score was lower in DRE compared to MCE (p<0.001). Also, QOLIE-31 subscores were significantly lower in terms of subdomains of seizure worry (p=0.001), overall quality of life (p=0.004), emotional well-being (p=0.005), and cognitive function (p=0.007) in DRE than in MCE.

**Table 3:** Comparison of clinical scales and heart rate variability parameters between medically controlled and drug-resistant epilepsy patients.

	MCE (n=27)	DRE (n=23)	p-value
HRV parameters			
SDNN (ms)	123 (102–159)	140 (111–166)	0.661
SDNN index	49.5 (40.5–64.5)	54 (46–66)	0.572
SDANN (ms)	110.5 (86.75–142.5)	110 (92–158)	0.661
RMSSD (ms)	29 (16.5–36.5)	30 (23–42)	0.335
pNN50 (%)	8 (2–15)	7 (4–18)	0.292
Minimum HR (b/m)	54.5 (48–57.25)	51 (45–55)	0.283
Maximum HR (b/m)	179 (137–248)	158 (128–220)	0.288
Average HR (b/m)	84 (76–97)	78 (70–85)	**0.048**
Maximum QT (ms)	489 (459–614)	475 (452–567)	0.307
Maximum QTc (ms)	537 (502–693)	544 (494–622)	0.645
PSQI	4 (2–6)	5 (4–6)	0.260
QOLIE-31 total score	68.2±16.6	51.0±11.9	**<0.001**
Seizure worry	74.7 (28.7–100)	30 (16.6–42.30)	**0.001**
Overall quality of life	64.0±23.0	45.6±18.7	**0.004**
Emotional well-being	66.2±16.6	51.0±20.1	**0.005**
Energy/fatigue	55.4±24.8	47.6±22.0	0.251
Cognition	71.8±23.5	54.5±19.4	**0.007**
Medication effects	91.7 (44.4–100)	66.70 (47.2–100)	0.164
Social function	71 (43–80)	75 (58–80)	0.339

Values are expressed as mean±SD or median (25th–75th percentile). MCE: medically controlled epilepsy, DRE: drug-resistant epilepsy, PSQI: Pittsburgh Sleep Quality Index, HRV: heart rate variability, SDNN: standard deviation of RR intervals, SDNN index: the standard deviations of all the NN intervals for each 5 min segment of a 24-h HRV recording, SDANN: the standard deviation of the average NN intervals for each of the 5 min segments, RMSSD: root mean square of the successive differences of inter-beat intervals, pNN50: percentage of adjacent NN intervals differing by more than 50 milliseconds, HR: heart rate, QTc: corrected QT, QOLIE-31: quality of life in epilepsy-31. p<0.05 was considered statistically significant.

When the correlations of HRV parameters with PSQI and QOLIE-31 were analyzed, there was a significant positive correlation between the SDANN and cognition (r=0.335; p=0.017); maximum QT and emotional well-being (r=0.286; p=0.046); maximum QTc and emotional well-being (r=0.292; p=0.042) in PWE.

## DISCUSSION

This study represents the first investigation into the relationship between HRV, quality of life, and sleep in PWE. In this study, we found that HRV parameters were lower in PWE compared to the control group. Also, HRV parameters were similar between DRE and MCE, while life quality was lower in DRE than in MCE. The correlation analysis revealed that some HRV parameters were positively associated with subdomains of cognition and emotional well-being. While prior research has linked HRV to SUDEP risk, our results suggest a broader clinical relevance of HRV as a biomarker for cognitive health and emotional stability in epilepsy.

Growing evidence has provided clues for a reduction in HRV in PWE compared to the normal population^9–11^. Altered parasympathetic autonomic activity during interictal periods causes a decrease in HRV^10^. It has been suggested that there may be progressive changes induced in autonomic centers by recurrent seizure discharges^9^. In line with the literature, we showed that HRV parameters (SDNN, SDNN index, SDANN, RMSSD, and pNN50) were lower in PWE than in controls, but those were similar between DRE and MCE, regardless of whether it was DRE or MCE. This means that all epileptic patients have disturbed HRV to some extent.

There are few studies on the relationship between HRV and life quality, but none on PWE. Kanbara et al. linked high HRV to better quality of life in functional somatic syndromes, noting that reduced vagal control lowers life quality^4^. Recent works have demonstrated that seizure frequency and/or severity, and psychiatric comorbidities are the most important factors affecting quality of life in PWE^1,12^. Duration of epilepsy and social and occupational difficulties were other risk factors^1,13^. A long disease, such as in DRE, can lead to a decline in the quality of life of these patients. Edefonti et al. reported that longer epilepsy duration worsens quality of life due to increased cognitive impairment^14^. HRV has been proposed as a potentially valuable early biomarker for cognitive impairment in individuals who do not present with medical conditions such as dementia, psychiatric disorders, strokes, or traumatic brain injuries^15^. Arakaki et al. highlighted a bi-directional heart-brain link, where reduced HRV impairs decision-making and emotional regulation^16^. Confirming this, we found some HRV parameters positively associated with subdomains of cognition and emotional well-being. Taken together, the data almost prove that lower HRV indicates lower vagal activity, indicating poor cognitive function^17^.

With respect to the relationship between HRV and sleep, vagal tonus is crucial in the induction and maintenance of sleep^18^. Sajjadieh et al. linked higher PSQI scores to lower HRV, concluding that poor sleep reduces HRV in their Iranian hospital study^19^. Another study by Burton et al. showed that HRV parameters were the best predictors of sleep quality in the multivariate analyses in patients with chronic fatigue syndrome^5^. Low HRV appears to correlate with a reduction in the vagal tone, which causes nocturnal sympathetic hyperactivity, disturbing sleep itself^5^. Differently, we explored the linkage between HRV with sleep in PWE. Contrary to expectations based on general population studies, our analysis did not reveal significant correlations between HRV parameters and sleep quality in PWE. As well, sleep quality was similar between DRE and MCE, although previous studies have reported that sleep quality is poorer in the DRE^20,21^. This may be due to the high use of lacosamide and valproic acid in DRE. Because lacosamide and valproic acid have been shown to improve PSQI scores and have a positive effect on sleep^22^. This suggests that pharmacological management may partially offset the adverse effects of autonomic dysregulation on sleep.

There are several limitations. First, the study was conducted at a single center and involved a small sample size. Second, it was cross-sectional. Third, cognition was evaluated as a subgroup of the quality of life scale, and detailed neuropsychometric tests were not performed. Fourth, video EEG monitoring was not conducted for seizure classification.

## CONCLUSION

Our study demonstrates that HRV is significantly reduced in PWE and correlates with specific domains of quality of life, particularly cognition and emotional well-being. Our results also showed that quality of life was highly impaired in DRE. These findings underscore the potential of HRV as a multidimensional biomarker in epilepsy management. A definitive answer may be obtained from future research with a large population.

## Data Availability

The datasets generated and/or analyzed during the current study are available from the corresponding author upon reasonable request.

## References

[1] Taylor RS, Sander JW, Taylor RJ, Baker GA (2011). Predictors of health-related quality of life and costs in adults with epilepsy: a systematic review. Epilepsia.

[2] Bergmann M, Tschiderer L, Stefani A, Heidbreder A, Willeit P, Högl B (2021). Sleep quality and daytime sleepiness in epilepsy: systematic review and meta-analysis of 25 studies including 8,196 individuals. Sleep Med Rev.

[3] Evangelista G, Dono F, Consoli S, Lanzone J, Corniello C, Russo M (2023). Heart rate variability modification as a predictive factor of sudden unexpected death in epilepsy: how far are we? A systematic review and meta-analysis. Eur J Neurol.

[4] Kanbara K, Morita Y, Hasuo H, Abe T (2021). The association between heart rate variability and quality of life in patients with functional somatic syndrome and healthy controls. Appl Psychophysiol Biofeedback.

[5] Burton AR, Rahman K, Kadota Y, Lloyd A, Vollmer-Conna U (2010). Reduced heart rate variability predicts poor sleep quality in a case-control study of chronic fatigue syndrome. Exp Brain Res.

[6] Kwan P, Arzimanoglou A, Berg AT, Brodie MJ, Allen Hauser W, Mathern G (2010). Definition of drug resistant epilepsy: consensus proposal by the ad hoc Task Force of the ILAE Commission on Therapeutic Strategies. Epilepsia.

[7] Vickrey B (1993). Quality of life in epilepsy (QOLIE 89). Scoring Manual and Patient Inventory.

[8] Buysse DJ, Reynolds CF, Monk TH, Berman SR, Kupfer DJ (1989). The Pittsburgh Sleep Quality Index: a new instrument for psychiatric practice and research. Psychiatry Res.

[9] Baysal-Kirac L, Serbest NG, Şahin E, Dede HÖ, Gürses C, Gökyiğit A (2017). Analysis of heart rate variability and risk factors for SUDEP in patients with drug-resistant epilepsy. Epilepsy Behav.

[10] Verrier RL, Pang TD, Nearing BD, Schachter SC (2021). Epileptic heart: a clinical syndromic approach. Epilepsia.

[11] Myers KA, Bello-Espinosa LE, Symonds JD, Zuberi SM, Clegg R, Sadleir LG (2018). Heart rate variability in epilepsy: a potential biomarker of sudden unexpected death in epilepsy risk. Epilepsia.

[12] Silva B, Canas-Simião H, Cordeiro S, Velosa A, Oliveira-Maia AJ, Barahona-Corrêa JB (2019). Determinants of quality of life in patients with drug-resistant focal epilepsy. Epilepsy Behav.

[13] Yogarajah M, Mula M (2019). Social cognition, psychiatric comorbidities, and quality of life in adults with epilepsy. Epilepsy Behav.

[14] Edefonti V, Bravi F, Turner K, Beghi E, Canevini MP, Ferraroni M (2011). Health-related quality of life in adults with epilepsy: the effect of age, age at onset and duration of epilepsy in a multicentre Italian study. BMC Neurol.

[15] Forte G, Favieri F, Casagrande M (2019). Heart rate variability and cognitive function: a systematic review. Front Neurosci.

[16] Arakaki X, Arechavala RJ, Choy EH, Bautista J, Bliss B, Molloy C (2023). The connection between heart rate variability (HRV), neurological health, and cognition: a literature review. Front Neurosci.

[17] Shaffer F, Ginsberg JP (2017). An overview of heart rate variability metrics and norms. Front Public Health.

[18] Borne P, Nguyen H, Biston P, Linkowski P, Degaute JP (1994). Effects of wake and sleep stages on the 24-h autonomic control of blood pressure and heart rate in recumbent men. Am J Physiol.

[19] Sajjadieh A, Shahsavari A, Safaei A, Penzel T, Schoebel C, Fietze I (2020). The association of sleep duration and quality with heart rate variability and blood pressure. Tanaffos.

[20] García-Morales I, Gil-Nagel A, Rosendo J, Torres-Falcón A (2014). [Sleep disorders and quality of life in refractory partial epilepsy: results of the SLEEP study]. Rev Neurol.

[21] Chakravarty K, Shukla G, Poornima S, Agarwal P, Gupta A, Mohammed A (2019). Effect of sleep quality on memory, executive function, and language performance in patients with refractory focal epilepsy and controlled epilepsy versus healthy controls - A prospective study. Epilepsy Behav.

[22] Liguori C, Toledo M, Kothare S (2021). Effects of anti-seizure medications on sleep architecture and daytime sleepiness in patients with epilepsy: a literature review. Sleep Med Rev.

